# A dynamic and adaptive class-balanced data augmentation approach for 3D LiDAR point clouds

**DOI:** 10.1371/journal.pone.0318888

**Published:** 2025-03-17

**Authors:** Bo Liu, Xiao Qi

**Affiliations:** 1 School of Computer Science and Artificial Intelligence, Chaohu University, Chaohu, China; 2 School of Computer Science and Engineering, Macau University of Science and Technology, Macau, China; 3 Department of Information Technology and Cybersecurity, Shanghai Police College, Shanghai, China; Purdue University, UNITED STATES OF AMERICA

## Abstract

3D LiDAR point clouds, obtained through scanning by LiDAR devices, contain rich information such as 3D coordinates (X, Y, Z), color, classification values, intensity values, and time. However, the original collected 3D LiDAR point clouds often exhibit significant disparities in instance counts, which can hinder the effectiveness of point cloud segmentation. PolarMix, a data augmentation algorithm for 3D LiDAR point cloud datasets, addresses this issue by rotating and pasting selected class instances around the Z axis multiple times to enrich the distribution of the point cloud. However, PolarMix does not adequately consider the substantial variations in instance counts within the original point clouds, leading to an imbalance in the dataset. To address this limitation, we propose a modified version of PolarMix’s instance-level rotation and pasting method that dynamically adjusts the number of rotations and pastes based on the proportion of each instance’s point cloud count relative to the total. This adaptive class-balancing approach ensures a more balanced distribution of instances across the entire dataset. We term our new algorithm Dynamic Adaptive Class-Balanced PolarMix (DACB-PolarMix). Experimental results demonstrate the effectiveness of DACB-PolarMix in balancing class distribution and enhancing model performance. The results on the SemanticKitti dataset are particularly significant. Under the MinkNet model, our method improved the mIoU from 65% to 67.9%, and under the SPVCNN model, our method increased the mIoU from 66.2% to 67.5%.

## Introduction

Advanced methodologies for data augmentation are pivotal in boosting the efficacy of semantic segmentation processes applied to three-dimensional (3D) Light Detection and Ranging (LiDAR) point cloud data. Data augmentation is a technique used in machine learning to artificially increase the diversity of a dataset by applying various transformations to the existing data. This process helps to improve the generalization ability of models by exposing them to a wider variety of examples during training. In datasets with imbalanced classes, data augmentation can be used to generate more samples for underrepresented classes, balancing the distribution.The output of data augmentation are transformed versions of the original data samples. In our study, the output of data augmentation involves replicating different object instances in point cloud data within their surrounding environment. The number of replications is inversely proportional to the number of original object instances.The augmented data is then used along with the original data to train machine learning models, resulting in a richer and more diverse training set.

**Fig 1:**
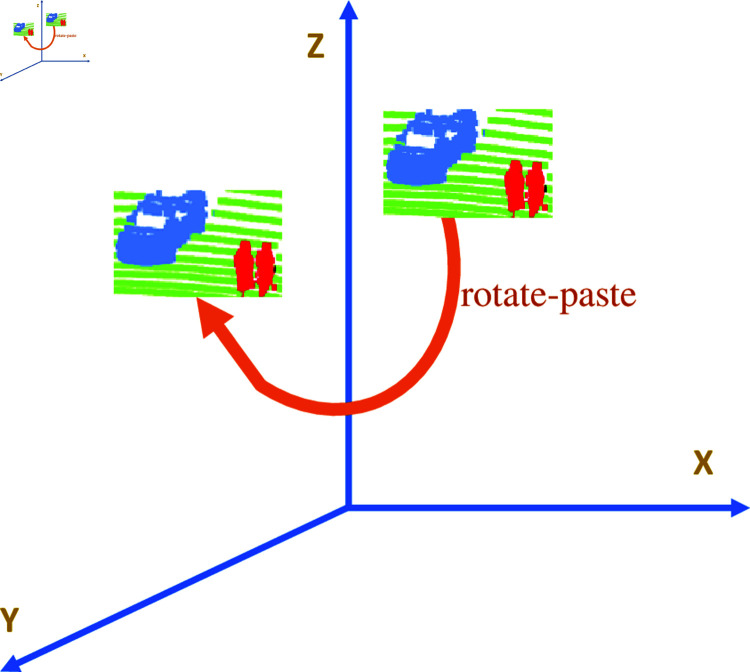
The illustration of instance-level operation of PolarMix.

A 3D LiDAR point cloud consists of millions of discrete data points that delineate the 3D coordinates of surfaces within the scanned area. This technology functions by emitting laser pulses and calculating the time taken for these pulses to rebound from an object, thereby capturing detailed geometric information about the shape, texture, and spatial relationships of objects within the scanned region [1,2]. Given their high resolution and precision, 3D LiDAR point clouds are essential resources in disciplines requiring meticulous spatial analysis and visualization. Innovative data augmentation strategies can substantially improve the accuracy, resilience, and overall effectiveness of segmentation algorithms [1–3]. Among these, the newly introduced PolarMix [3] approach marks a significant advancement in the domain of enhancing 3D LiDAR Point Cloud Semantic Segmentation,it involves cropping point instances from a single LiDAR scan, rotating these instances at multiple angles to create multiple copies, and finally pasting these rotated instances into other scans. However, the number of times this operation is performed is the same for all classes. A notable drawback of the PolarMix method is its uniform application of the rotate-paste procedure to class instances, which does not address the issue of class imbalance. Fig 1 shows the instance-level rotate-paste operation of PolarMix.

**Fig 2:**
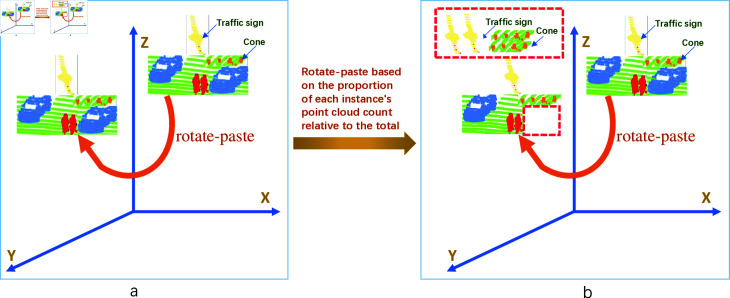
The illustration of the core idea of DACB-PolarMix.

To address the issue of class imbalance, we propose the DACB-PolarMix methodology. Fig 2 elucidates the fundamental concept behind DACB-PolarMix. In the conventional PolarMix approach, during the instance-level rotate-paste operations, every instance undergoes the same number of rotations and pastes, as depicted on the left side of Fig 2. This means that cars, persons, traffic signs, and cones all experience an equal number of rotate-paste iterations. Conversely, DACB-PolarMix adaptively modifies the frequency of rotate-pasting for each category according to their representation in the initial dataset. As illustrated on the right side of Figure 2, cars undergo only one rotate-paste, traffic signs receive an additional rotate-paste to total three times, and cones are subjected to two more rotate-pastes, culminating in three instances. Consequently, each class is rotate-pasted a distinct number of times.

The primary contributions of this study are twofold:

(1) Dynamic Adaptive Class-Balanced PolarMix: We introduce a novel adaptation of PolarMix’s instance-level rotation and pasting technique, which we call Dynamic Adaptive Class-Balanced PolarMix (DACB-PolarMix). This method dynamically modulates the extent of rotations and pasting operations based on the proportional representation of each instance’s point cloud count in relation to the aggregate dataset. By doing so, it achieves an improved equilibrium in the distribution of instances throughout the dataset, thereby enhancing class balance adaptively.

(2) Enhanced Performance: Utilizing the models of MinkowskiNet [4] and SPVCNN [5], our experimental investigations were extended to encompass both the semanticKitti [6] and semanticPOSS [7] datasets. The findings from these comprehensive tests indicate a consistent superiority of our introduced DACB-PolarMix approach over the conventional PolarMix methodology across all evaluated scenarios.

## Related work

### Current status of 3D LiDAR point cloud semantic segmentation

The progression of 3D LiDAR point cloud semantic segmentation can be traced back to the incorporation of deep learning methodologies [8]. Historically, methods that relied heavily on manually crafted features and geometric analysis have been steadily supplanted by neural network-based architectures capable of extracting and utilizing high-dimensional features from raw point cloud data. A significant milestone in this domain was the introduction of PointNet [9], which directly processes unordered point clouds using a shared Multi-Layer Perceptron (MLP) and a max-pooling layer to capture global features. This groundbreaking work paved the way for subsequent architectures such as PointNet++ [10], which incorporated hierarchical feature learning to capture local geometric structures. Other notable architectures include PointCNN [11], which utilizes a permutation-invariant convolution operation to process point clouds, and KPConv [12], which employs kernel points to define convolutional weights, allowing for efficient feature extraction from sparse point clouds.

Despite the progress made, several challenges remain in theDespite the progress made, several challenges remain in the LiDAR point cloud several challenges remain in the field of 3D LiDAR point cloud semantic segmentation. A major obstacle is the high computational expense involved in handling large-scale point cloud data [13]. Strategies like voxelization, which transforms point clouds into a more manageable voxel grid, have been implemented to address this issue, but they often result in information loss. Additionally, the irregular and sparse nature of point cloud data presents difficulties for traditional convolutional neural networks (CNNs), which are designed for structured grid data. This has led to the creation of specialized architectures that can handle point clouds more effectively, but these architectures often require significant computational resources. Furthermore, annotating 3D point cloud data is labor-intensive and time-consuming, limiting the availability of large-scale labeled datasets. This scarcity of labeled data hinders the training of deep learning models and their ability to generalize to new, unseen environments.

To address these challenges, researchers are exploring various strategies. One promising direction is the development of more efficient and lightweight architectures that can process large-scale point cloud data with reduced computational resources. Another approach is the use of unsupervised and semi-supervised learning techniques to leverage unlabeled data and reduce the reliance on labeled datasets. Additionally, integrating multi-modal information, such as RGB images and LiDAR scans, has shown potential in improving the accuracy and robustness of 3D point cloud semantic segmentation models. As LiDAR sensors continue to evolve and become more affordable, the availability of high-quality 3D point cloud data will increase, further driving advancements in this field.

### Data augmentation techniques in 3D LiDAR point cloud semantic segmentation

A foundamental study on improving 3D object detection through data augmentation using LiDAR data is detailed in [14]. This research comprehensively examined a variety of global and local augmentation strategies, all implemented within the context of a voxel-centric 3D object detection framework named PointPillars. Global augmentation techniques, which involve transformations applied to the entire point cloud scene, include operations such as global translation, rotation, and scaling. On the other hand, local augmentation techniques focus on individual objects within the scene, applying transformations like translation, rotation, and jittering. Their findings revealed that both types of augmentation can lead to performance improvements, by applying their findings to the PointPillars data augmentation policy, the authors achieved a performance increase of up to 2%. Another significant advancement in data augmentation for point cloud semantic segmentation is the introduction of geometric and semantic context enhancement techniques. These innovations aim to clarify the distinction between closely spaced points and provide a more comprehensive interpretation of the immediate surroundings. In reference [15], the researchers introduced a dual-augmentation strategy that leverages both geometrical and semantic attributes to enrich local contextual information of points. This approach utilizes a bilateral context module, which integrates data from various resolutions and adaptively incorporates multi-scale features, resulting in improved accuracy in semantic segmentation. The bilateral context module comprises a series of bilateral context blocks, which refine the local context for each point by incorporating offsets derived from semantic cues.

The evolution of data augmentation techniques for 3D LiDAR point clouds has paralleled advancements in both voxel-based and point-based methodologies for 3D object detection and semantic segmentation. Initial voxel-based methods, such as VoxelNet [16], faced challenges due to high memory usage associated with 3D convolutional neural networks (CNNs). To address this, sparse convolutional networks (SparseConvNet) [17] were introduced, significantly reducing memory consumption while enabling finer voxel resolutions. Subsequent approaches, like PointPillars [18], optimized voxel-based methods by converting voxels into pillars, eliminating the need for 3D convolutions.

Recent studies have explored hybrid approaches that leverage the strengths of both voxel-based and point-based methods. A notable example is PV-RCNN [19], which combines voxel-based feature encoding with point-based feature aggregation for superior 3D object detection performance. The approach uses a voxel feature encoding (VFE) layer followed by a point-based set abstraction (SA) layer to aggregate features from neighboring points. By integrating voxel-based efficiency with point-based accuracy, PV-RCNN outperforms previous methods. Data augmentation in hybrid methods often involves global and local transformations tailored for both voxel-based and point-based components. For instance, voxel-based augmentations may include random scaling and flipping of voxel grids, while point-based augmentations might involve random rotations and translations of individual points. Carefully designed data augmentation strategies for hybrid models can further enhance their performance and generalization capabilities.

Self-supervised learning has emerged as a promising direction for utilizing unlabeled data to enhance deep learning model performance. In the context of 3D LiDAR point clouds, self-supervised learning can generate pseudo-labels for unlabeled data, augmenting the training set. An example of self-supervised learning for 3D point clouds is predicting the relative poses of overlapping point clouds [20]. Training a model to predict these transformations generates additional labeled data, improving semantic segmentation model performance.

Another category of data augmentation techniques employs some form of “mixing” or “cutting” strategy to create new point cloud data samples. For instance, the PointMixUp [21] method generates new point clouds by interpolating points between pairs of point clouds. Each point in the newly created point cloud is a weighted combination of points from the two original point clouds, with the weights randomly sampled from a beta distribution. This approach not only blends the coordinates of the point clouds but also interpolates the associated features and labels. On the other hand, the PointCutMix [22] method performs “cutting and mixing” on point clouds, analogous to the CutMix technique used in image data. It randomly replaces a portion of points (which can be a random sample, a spherical region, or any other defined shape) in one point cloud with points from another point cloud. The labels are also mixed accordingly, retaining the original label if the point is from the first point cloud and adopting the label from the second point cloud if it is from the latter. The GT-Aug [23] method is specifically designed for object detection tasks, enhancing data by cutting out instances and integrating them into other LiDAR scans. This requires 3D bounding boxes for object segmentation. The Mix3D [24]method creates new 3D samples by mixing two existing samples. It blends not only the features of the points (such as coordinates and other attributes) but also the labels associated with each point or 3D object. Each point (or voxel, in the case of volumetric data) in the new sample is a weighted average of the corresponding points in the two original samples, with the weights typically randomly chosen from a distribution, such as the beta distribution.These techniques collectively contribute to generating new training samples that combine features and labels from original samples but exhibit greater diversity and variability. Such data augmentation strategies aid models in learning more robust feature representations, improving their generalization capabilities on unseen data, and reducing the risk of overfitting.

### Newly emerged 3D point cloud datasets

Advances in deep learning-based 3D vision, especially in NeRF and NVS applications, have been constrained by limited diverse scene-level datasets. Existing datasets are either synthetic or lack real-world variety, which hinders comprehensive benchmarking. The introduction of the DL3DV-10K dataset [25], comprising 51.2 million frames from 10,510 videos across 65 POI locations, has enabled detailed benchmarking of recent NVS methods. This provides critical insights for future research and underscores the need for large-scale datasets in developing foundational 3D models. In autonomous driving, perception models benefit from large-scale point-cloud datasets that allow for unified representations across tasks. A new large-scale pre-training point-cloud dataset [26] with diverse data distribution was constructed to address this need. Additionally, a semi-supervised pre-training approach leveraging both few-shot labeled and massive unlabeled data has shown significant performance gains in benchmarks like Waymo, nuScenes, and KITTI. Moreover, the expansion of LiDAR technology to forest monitoring highlights the importance of understanding point distribution. The LiDAR-Forest dataset [27] and simulation tool support low-cost research in forest reconstruction and tree DBH estimation. For concrete bridge surface defect detection, a new publicly available dataset [28] combined with the Surface Normal Enhanced PointNet++ (SNEPointNet++) model addresses challenges related to defect complexity. This approach achieves high recall for detecting cracks, spalls, and severe spalls.

## Proposed method: DACB-PolarMix

Dynamic Adaptive Class-Balanced PolarMix is based on the instance-level rotate-paste operation of PolarMix. It dynamically adapts the number of times the rotate-paste operation is performed based on the proportion of point cloud instances belonging to a certain class relative to the total number of point clouds. The lower the proportion, the higher the number of rotate-paste operations. This approach achieves balance among different classes in the point cloud data.

### Preliminaries

The dataset generated by a 3D LiDAR sensor is referred to as a 3D LiDAR point cloud. These sophisticated sensors function by emitting laser pulses towards surrounding objects, with the time it takes for these pulses to return after striking an object enabling the sensor to determine the precise distance to each object. Consequently, this data is depicted as a cluster of 3D points, with each point signifying a distinct spatial location. Such detailed and high-resolution data is essential for applications such as autonomous driving, robotics, and geographic mapping.

**Table 1:** Correspondence between classes and colors in the SemanticPOSS dataset.

class numbers	colors (RGB)	colors
0	[255, 30, 30]	Bright Red
1	[255, 40, 200]	Light Pink
2	[100, 150, 245]	Light Blue
3	[135, 60, 0]	Dark Brown
4	[0, 175, 0]	Green
5	[255, 0, 0]	Red
6	[255, 240, 150]	Pale Yellow
7	[50, 255, 255]	Aqua
8	[255, 200, 0]	Orange
9	[255, 150, 0]	Orange
10	[255, 120, 50]	Light Orange
11	[100, 230, 245]	Light Blue
12	[150, 240, 80]	Light Green
23	[0, 0, 0]	Black

The field of 3D point cloud technology has been rapidly advancing, with numerous 3D LiDAR point cloud datasets emerging. SemanticKITTI and SemanticPOSS are two prominent datasets used in the field of 3D point cloud semantic segmentation tasks. The SemanticKITTI dataset was introduced as an extension of the well-known KITTI dataset, which has long been a benchmark for computer vision research in autonomous driving and 3D scene understanding. The original KITTI dataset, released by the Karlsruhe Institute of Technology (KIT) and Toyota Technological Institute (TTI), provided a comprehensive collection of stereo images, point clouds, and various sensor data captured from a car driving through urban and rural environments. Recognizing the need for more advanced semantic information, researchers developed the SemanticKITTI dataset. This enhanced version includes detailed annotations that categorize each point in the point clouds into specific classes such as cars, pedestrians, cyclists, buildings, and other environmental elements. This additional layer of semantic information has significantly boosted the research in 3D object detection and semantic segmentation. While less widely known than the SemanticKITTI dataset, the SemanticPOSS dataset has also contributed significantly to the field of 3D point cloud semantic segmentation. The POSS dataset itself was created to provide high-quality 3D point cloud data with precise annotations for research purposes. The semantic variant of this dataset builds upon the foundational work by incorporating detailed semantic labels into the point clouds.

**Fig 3:**
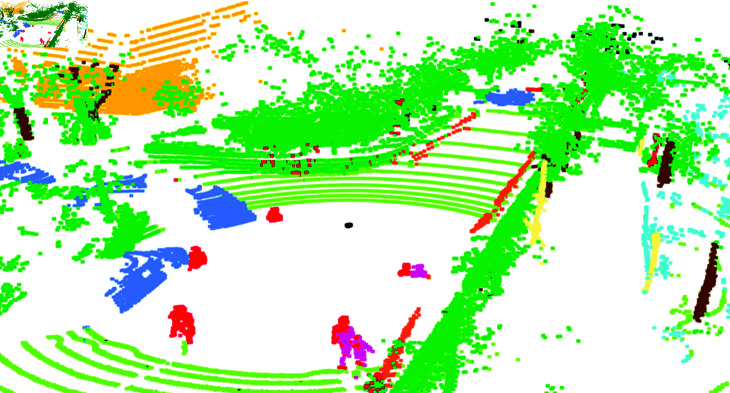
The visualization of a 3D point cloud.

To provide a clear visualization of our experimental results, we have assigned unique colors to each class in the SemanticPOSS dataset, as presented in Table 1. These color assignments are consistent with the official standards set by the respective datasets. This color coding facilitates an intuitive comparison between the predicted point cloud and the actual ground truth during the 3D point cloud data visualization process. The color of each point acts as a simple indicator to evaluate the accuracy of semantic segmentation predictions. For example, in Fig 3, which is a screenshot from scan 000001 of sequence 03 in the SemanticPOSS dataset, the color codes help us easily identify different entities in the scene, such as red for persons, green for plants, blue for cars, and brown for tree trunks, among others. Thus, the use of color not only improves the visual appeal but also aids in better understanding the segmentation outcomes.

In our investigation of PolarMix, we observed that the process of rotating and pasting selected object instances is uniformly applied; every chosen instance undergoes an identical sequence of rotations and pasting operations. While it is plausible that theoretically, repeating these actions could amplify the quantity of instances within a single LiDAR scan frame, thereby enriching the dataset for model training, we contend that adopting a uniform approach to pasting across all instances may not be the optimal strategy for boosting the accuracy of semantic segmentation tasks.

Upon reviewing the initial semantic segmentation outcomes from PolarMix, we observed a substantial variation in accuracy across distinct object categories. Specifically, when employing MinkowskiNet on the SemanticKITTI dataset, classes such as cars, roads, and buildings exhibited segmentation accuracies exceeding 80%, with some even surpassing 90%. Conversely, categories like motorcyclists and other ground surfaces lagged behind, with accuracies hovering around a mere 10%. This glaring discrepancy in segmentation precision across different semantic classes led us to consider modifications to the original PolarMix framework. To address this imbalance, we introduce a strategy that dynamically adjusts the representation of each class within the training dataset. By augmenting the frequency of certain classes through repeated rotate-and-paste operations, we aim to elevate their prominence in the dataset. The extent of this augmentation is directly proportional to the underrepresentation of each class in the original dataset; the less frequent a class appears, the more it is subjected to the rotate-paste procedure. This targeted approach serves to recalibrate the class distribution, ensuring a more equitable learning process for all categories. Ultimately, this technique seeks to bolster the semantic segmentation performance for underrepresented classes, thereby elevating the overall model accuracy when applied to specific datasets. By fine-tuning the balance of class representations, we anticipate significant improvements in the model’s ability to accurately discern and segment diverse objects within complex scenes.

For the segmentation of 3D LIDAR point clouds models, we employed MinkowskiNet and Sparse Voxel Convolutional Neural Networks (SPVCNN). MinkowskiNet represents a specialized neural network architecture tailored for the processing of sparse, high-dimensional datasets, frequently observed in domains such as robotics, autonomous driving, and 3D (3D) vision. Its nomenclature draws inspiration from Minkowski spacetime, a mathematical construct employed in physics to depict spatiotemporal relationships. However, within the realm of neural networks, it signifies the Minkowski Engine—a vital mechanism facilitating the adept management of sparse tensor inputs. This network employs an advanced form of convolution meticulously adapted for handling high-dimensional, sparse information. In contrast to conventional convolutional neural networks (CNNs), which operate under the assumption of data density akin to image pixels, MinkowskiNet is ingeniously crafted to accommodate scenarios where the majority of the input space remains unoccupied—a hallmark characteristic of point cloud data. By concentrating solely on populated data points, MinkowskiNet circumvents superfluous computations associated with vacant regions, thereby significantly enhancing computational efficacy and optimizing memory utilization. Sparse Voxel Convolutional Neural Networks (SPVCNN) constitute another variant of 3D CNNs specifically engineered to tackle point cloud data challenges. SPVCNN distinguishes itself through its proficiency in managing sparse 3D datasets. Conventional 3D CNNs typically grapple with the substantial computational and memory overhead necessitated by extensive volumes of empty or insignificant data. To address this predicament, SPVCNN implements voxelization, partitioning the 3D expanse into discrete units known as voxels. Subsequently, it selectively processes only those voxels harboring pertinent information, drastically diminishing the computational burden and resource consumption.

### Algorithm of DACB-PolarMix

Dynamic Adaptive Class-Balanced PolarMix is built upon the instance-level rotate-paste operation of PolarMix. It dynamically adjusts the frequency of the rotate-paste operation based on the proportion of point cloud instances pertaining to a specific class relative to the total number of point clouds. The lower this proportion, the more frequent the rotate-paste operations become. This modification aims to increase the representation of underrepresented classes in the dataset, thereby enhancing their learning by the model and subsequently improving the performance of point cloud segmentation tasks.

Regarding the specific algorithm of DACB-PolarMix, we offer a comprehensive explanation accompanied by the pseudocode provided. This pseudocode outlines a method for dynamic adaptive class-balanced PolarMix, similar to its implementation in PyTorch. Initially, it calculates the total number of points present in the labels and initializes a count array for each instance class. As it iterates through each label, it checks if the label exists within the label map dictionary. If found, the label is mapped to its corresponding value, and if this value belongs to the instance classes, the count for that class is incremented. Following this counting process, proportions are determined by dividing counts by the total points and normalized by dividing by the sum of all proportions. Based on these normalized proportions, rotation counts are established, scaled by a factor, and adjusted to stay within predefined maximum and minimum bounds. Finally, the rotation counts are clipped and converted into integers before being returned. This approach ensures that the frequency of rotations during data augmentation is dynamically tailored to the distribution of classes, thus promoting balance and enhancing model performance. Based on the pseudocode, we calculated the computational efficiency of our proposed method. We considered the time complexity of each step in the pseudocode and then obtained the overall time complexity, which can be approximated as: O(max(m, n)) where n is the number of unique instance classes, and m is the length of labels.

**Table:** 

Algorithm 1: Pseudocode of Dynamic Adaptive class-balanced Polarmix Method ina PyTorch-like style.	
1:	#instance_classes: the object instances required for ratation operations in the DACB-Polarmix Method.
2:	#label_map_dict: A dictionary maps integer keys to integer values, which represent classes of objects within a dataset.
3:	# Calculate the total number of points in labels
4:	total_points = len(labels)
5:	# Initialize an array to count occurrences of each instance_class
6:	counts = np.zeros(len(instance_classes))
7:	# Iterate through each label in labels
8:	for label in labels:
9:	# Check if the label is in the label_map_dict
10:	if label in label_map_dict:
11:	# Map the label to its corresponding value
12:	mapped_label = label_map_dict[label]
13:	# Check if the mapped label is in instance_classes
14:	if mapped_label in instance_classes:
15:	# Increment the count at the index of the mapped label in counts
16:	counts[instance_classes.index(mapped_label)] += 1
17:	
18:	# Calculate proportions by dividing counts by total_points
19:	proportions = counts / total_points
20:	# Normalize the proportions by dividing each element by the sum of all elements in proportions
21:	normalized_proportions = proportions / proportions.sum()
22:	
23:	# Set maximum and minimum rotation counts
24:	max_count = 3
25:	min_count = 1
26:	# Calculate rotation counts based on normalized proportions
27:	rotation_counts = max_count - (normalized_proportions * 10)
28:	# Clip rotation counts between min_count and max_count, converting them to integers
29:	rotation_counts = np.clip(rotation_counts, min_count, max_count).astype(int)
30:	
31:	# Return the calculated rotation counts
32:	return rotation_counts

## Experiments

### Datasets preprocessing

We conducted experiments using both the SemanticKITTI and SemanticPOSS datasets. For SemanticKITTI, we utilized a standard approach of evaluating with 19 semantic classes, aligning with methods used by other researchers. The SemanticKITTI dataset consists of 22 road sequences, labeled from sequence 00 to sequence 21. Following common practices, sequence 08 was designated as the validation set, while sequences 00-07, 09, and 10 constituted the training set. In line with the original PolarMix paper, we selected instances from classes 0 to 7 for rotation and pasting, applying an equal number of pastes across all classes.

The SemanticPOSS dataset, collected at Peking University, encompasses 6 road sequences, labeled as 00 to 05, and includes a total of 2988 diverse LIDAR scans in the same data format as SemanticKITTI. Consistent with standard practices, sequence 03 served as the validation set, while sequences 00, 01, 02, 04, and 05 composed the training set. This dataset involves 17 classes in total. In our experiments, we remapped these 17 classes to 14, as detailed in Table 2 . The “unlabeled” class was excluded from our experiments, and we concentrated on the evaluation of the remaining 13 classes. Regarding the SemanticPOSS dataset, we opted for the original PolarMix approach, selecting instances from classes 0, 1, 2, 5, 6, 7, 9, and 11 for rotation and pasting, with each class receiving equal pasting instances.

**Table 2:** Mapping relationship for remapping the SemanticPOSS dataset from 17 classes to 14 classes.

old class numbers	new class numbers	labels
0	0	1 person
4	0	2+ person
5	1	rider
6	2	car
7	3	trunk
8	4	plants
9	5	traffic sign 1(standing sign)
10	5	traffic sign 2(hanging sign)
11	5	traffic sign (high/big hanging sign
12	6	pole
13	7	trashcan
14	8	building
15	9	cone/stone
16	10	fence
17	11	bike
21	12	ground
22	23	unlabeled

### Hyperparameter setting and training

Concerning the configurations for rotation angles and paste durations, during the initial phase of rotation and application, we adhered to the parameters established in the foundational PolarMix document, which were specified as follows:

$\omega_{0}=\left[\frac{2\pi }{3}\times \text{np.random.random()},\frac{2\pi }{3}\times (\text{np.random.random()}+1)\right]$

For the number of rotate-paste operations, we set the maximum number of rotate-pastes to 3 and the minimum number of rotate-pastes to 1. As for the training and testing environments, on Ubuntu, Python version 3.8 is used, Pytorch version 1.6.0 is employed, the optimizer is set to Stochastic Gradient Descent (SGD), the learning rate is set to 0.24, momentum is set to 0.9, weight decay is set to 0.0001, and the number of training epochs is set to 55. Table 3 shows the aforementioned environmental hyperparameter settings.

**Table 3:** Hyperparameter setting.

Item	Version
Python	3.8
ubuntu	18.04
PyTorch	1.6.0
Rotate-paste max count	3
Rotate-paste min count	1
learning rate	0.24
optimizer	Stochastic Gradient Descent(SGD)
epoch	55
momentum	0.9
weight decay	0.0001

## Experiment results and discussion

### Evaluation metrics

In the realm of 3D LIDAR point cloud segmentation, the Intersection over Union (IoU) metric plays a pivotal role in assessing the fidelity and accuracy of a model’s predictive output relative to the ground truth. IoU quantifies the extent to which the predicted segmentation overlaps with the actual one by calculating the ratio between the intersection and the union of these two sets. This measurement is especially pertinent when evaluating the efficacy of semantic segmentation models, whose objective is to categorize each point within the point cloud into specific classes, such as ground, buildings, trees, etc. An IoU score ranges from zero to one; a higher value signifies greater overlap, thereby denoting a more precise model prediction.

Building upon this foundational concept, the Mean Intersection over Union (mIoU) extends the utility of IoU by offering a holistic assessment of a model’s performance across multiple categories. mIoU is derived by computing the average IoU for each category individually. This aggregated metric is crucial in contexts where the point cloud must be divided into several distinct classes, providing an overall perspective on the model’s performance. For example, if a point cloud segmentation task involves distinguishing among three categories—ground, buildings, and trees—the mIoU would be obtained by first determining the IoU for each category and then averaging these values. A superior mIoU score suggests that the model consistently performs well across all categories, highlighting its robustness in accurately classifying points. In practical terms, mIoU serves as a benchmark for comparing various models, informing the refinement and advancement of segmentation algorithms, and ensuring that the final model meets the necessary precision standards for real-world applications.

### Comparison with state-of-the-art

Table 4 presents the outcomes of a comparative experiment between PolarMix and our proposed DACB-PolarMix. The dataset employed is SemanticPOSS, while the model used is MinkowskiNet. The experiment involved rotating and pasting operations on classes 0, 1, 2, 5, 6, 7, 9, and 11. Test results for PolarMix are shown in the +PolarMix column, and those for DACB-PolarMix are presented in the +DACB-PolarMix column. From the final mIoU perspective, while the advantage may not be substantial, the DACB-PolarMix algorithm still demonstrates superior performance.

**Table 4:** Comparison of results based on SemanticPOSS dataset and MinkowskiNet Model.

class	+PolarMix	+DACB-PolarMix (ours)
person	61.6	61.6
rider	65.6	66.1
car	77.3	74.1
truck	33.1	30.6
trunk	78.5	78.4
traffic sign	47.5	50.2
pole	41.2	41.4
trashcan	39.0	37.2
building	79.6	80.2
cone /stone	42.2	46.8
fence	63.3	63.4
bike	54.8	56.0
ground	80.6	80.6
mIoU	58.8	59.0

Table 5 presents the outcomes of another comparative experiment between PolarMix and our proposed method, DACB-PolarMix. The experimental setup involves the use of the SemanticKitti dataset and the MinkowskiNet model. Initial steps involved applying rotation and pasting transformations to specific classes: 0, 1, 2, 5, 6, 7, 9, and 11. Following this, training was conducted using both PolarMix and DACB-PolarMix. Test results for PolarMix are shown in the +PolarMix column, while those for DACB-PolarMix are presented in the +DACB-PolarMix column. Observing the mIoU results, it can be seen that the metric improved from 57.7 to 58.2 with the implementation of DACB-PolarMix, clearly demonstrating that DACB-PolarMix outperforms PolarMix in this context.

**Table 5:** Comparison of results based on SemanticPOSS dataset and SPVCNN Model.

class	+PolarMix	+DACB-PolarMix (ours)
person	59.0	57.9
rider	63.9	64.7
car	66.2	63.1
truck	31.1	38.6
trunk	76.5	77.3
traffic sign	54.2	51.2
pole	45.8	45.2
trashcan	43.3	43.1
building	76.8	77.6
cone /stone	39.8	42.8
fence	61.2	61.6
bike	52.5	53.3
ground	79.9	80.4
mIoU	57.7	58.2

Table 6 presents the outcomes of the experimental evaluation conducted on the SemantiKitti dataset. The data for the DACB-PolarMix approach are derived from our in-house experiments, whereas the results pertaining to alternative methodologies are sourced from reference [3]. The table is bifurcated into two segments; the upper segment details the findings related to the MinkowskiNet architecture. Initially, operations involving rotation and pasting were applied to classes 0 through 7. Post-training assessments utilizing the PolarMix algorithm are reflected in the +PolarMix entry, whereas the corresponding figures for DACB-PolarMix are documented under the +DACB-PolarMix heading. Examination of the mean Intersection over Union (mIoU) scores reveals an enhancement from 65.0 to 67.9 upon integrating DACB-PolarMix, signifying marked progress. The subsequent section of Table 6 illustrates the experimental outcomes associated with the SPVCNN framework. Paralleling the prior procedure, rotation and pasting manipulations were initially executed across classes 0 to 7. Outcomes post-algorithmic training with SPVCNN in conjunction with the PolarMix technique are exhibited within the +PolarMix row, with those achieved through DACB-PolarMix featured in the +DACB-PolarMix row. Examination of the mIoU metrics discloses an uplift from 66.2 to 67.5 following the adoption of DACB-PolarMix, further affirming substantial advancement.

**Table 6:** Comparison of experimental results based on SemanticKitti dataset.

classes	MinkNet	+CGA	+CutMix	+CopyPaste	+Mix3D	+PolarMix	ours		SPVCNN	+CGA	+CutMix	+CopyPaste	+Mix3D	+PolarMix	ours
car	95.9	96.3	96.0	96.6	96.3	96.3	97.1		94.9	96.1	96.1	96.0	96.5	96.5	97.0
bicycle	3.7	8.7	10.2	18.4	29.6	51.2	54.0		9.1	21.8	21.4	32.4	35.9	53.9	56.9
motorcycle	44.9	52.3	59.3	62.8	61.8	75.6	78.5		55.8	57.8	59.6	66.4	65.0	79.7	81.6
truck	53.2	63.2	78.7	76.3	68.5	63.4	76.3		66.5	69.2	71.2	67.1	66.6	68.5	72.7
other-vehicle	42.1	51.6	52.1	64.6	55.4	63.9	74.2		33.7	49.8	54.2	52.9	60.2	64.9	71.0
person	53.7	63.5	63.4	68.9	72.7	71.9	73.2		61.8	66.7	66.8	74.8	75.3	75.6	73.6
bicyclist	68.9	74.4	79.4	82.8	77.7	85.6	89.4		75.9	80.8	81.8	84.3	83.3	87.8	88.3
motorcyclist	0.0	0.1	0.0	1.0	1.0	4.9	21.9		0.2	0.0	0.0	3.6	0.0	7.5	18.1
road	92.8	93.3	93.5	93.1	94.3	93.6	94.1		93.1	93.4	93.5	93.3	93.8	93.5	93.7
parking	43.0	46.6	47.8	45.3	52.9	45.8	51.8		45.3	44.8	49.6	46.9	49.0	47.3	47.1
sidewalk	80.0	80.4	80.7	80.2	81.7	81.4	82.3		79.6	80.1	81.1	80.2	81.1	81.2	81.4
other-ground	1.8	0.8	1.6	1.4	0.9	1.4	2.8		0.4	0.2	2.2	2.5	1.4	1.1	1.6
building	90.5	90.3	90.3	90.5	89.1	91.0	90.6		91.4	90.9	90.9	91.1	90.6	91.2	90.8
fence	60.0	60.0	61.0	60.7	55.5	62.8	60.6		62.7	62.9	63.1	64.1	60.0	63.8	61.6
vegetation	87.4	88.0	87.5	88.1	88.3	88.4	87.6		87.5	88.5	87.9	88.1	89.2	88.2	87.5
trunk	64.5	65.1	66.2	67.8	69.3	68.5	67.0		66.2	64.8	66.9	67.0	70.2	68.2	69.3
terrain	73.3	74.5	73.3	74.6	74.6	75.0	73.1		72.9	75.7	74.1	73.9	76.4	74.2	72.4
pole	62.1	62.8	64.0	63.7	65.2	64.6	64.6		62.8	63.6	63.8	64.0	64.8	64.5	64.7
traffic-sign	43.7	46.8	46.8	49.1	50.3	49.9	50.9		42.7	46.2	49.8	51.6	50.5	49.4	53.2
mIoU	55.9	58.9	60.6	62.4	62.4	65.0	67.9		58.0	60.7	61.7	63.2	63.7	66.2	67.5

**Fig 4:**
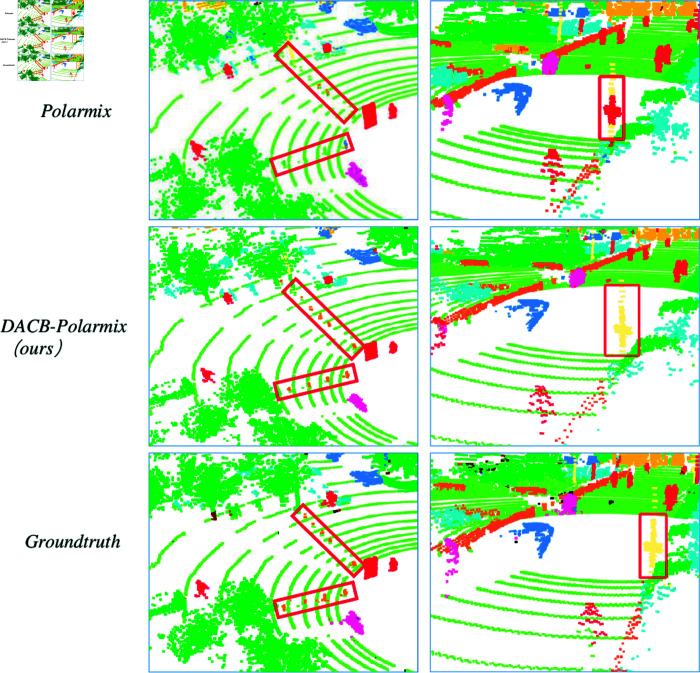
Visualization of comparative experiment results. The content within the red line box demonstrates the various performances of semantic segmentation.

The graphical representation in Fig 4 illustrates the empirical findings derived from implementing the MinkowskiNet framework on the SemantiPOSS dataset. For a comprehensive analysis, two distinct subsets of visualized data have been chosen: the initial set originates from sequence 000109, which is the third sequence within the SemanticPOSS dataset, and the second set comes from sequence 000411, also part of the third sequence in the SemanticPOSS dataset. Each subset of visualization data is organized into a separate column to facilitate side-by-side comparison and detailed examination, with every row displaying the outcomes for PolarMix, DACB-PolarMix, and the Ground truth. In examining the first column, it becomes clear that PolarMix failed to detect cones (highlighted by red lines), whereas DACB-PolarMix accurately identified these cones. Turning to the second column, PolarMix predominantly miscategorized the majority of traffic signs (encircled in red lines) as a red person, in contrast to DACB-PolarMix, which successfully recognized the traffic sign. These visual results serve as a compelling testament to the efficacy of DACB-PolarMix.

## Conclusion

In this study, we tackle the challenge of imbalanced instance counts in 3D LiDAR point cloud datasets, which significantly hinders effective point cloud segmentation. We introduce a novel algorithm, Dynamic Adaptive Class-Balanced PolarMix (DACB-PolarMix), that extends the existing PolarMix method by dynamically adjusting the number of rotation and pasting operations based on the proportion of instances in each class. This adaptive approach ensures a more balanced distribution of instances across the entire dataset, thereby improving the overall performance of 3D point cloud instance segmentation models.

However, it is important to acknowledge certain limitations of the proposed approach. While DACB-PolarMix effectively balances the class distribution, it may introduce computational overhead due to the dynamic adjustment of augmentation levels. This could potentially affect the scalability of the method for very large datasets. Additionally, the increased complexity may pose challenges in real-time applications where computational resources are limited. Future work can explore the application of DACB-PolarMix on more up-to-date datasets and consider the generalizability of our proposed method. There is also room for optimizing the algorithm to reduce computational costs while maintaining its effectiveness.

In summary, DACB-PolarMix represents a significant advancement over traditional data augmentation techniques for 3D LiDAR point clouds. By dynamically adapting the level of augmentation based on class proportions, our method achieves a more balanced dataset, ultimately contributing to better performance of 3D point cloud segmentation models. This work underscores the importance of addressing class imbalance in 3D LiDAR point clouds and provides a practical solution that can be readily adopted in various applications involving 3D point cloud analysis.
